# Juxta-Cortical Chondroma of the Phalanges: Is there a Role for Cone-Beam Computed Tomography in Diagnosis and Local Staging? Main teaching point: Low-dose cone-beam computed tomography (CT) may be of additional value to radiographs and magnetic resonance imaging (MRI) in preoperative characterization and local staging of juxta-cortical chondroma

**DOI:** 10.5334/jbsr.1657

**Published:** 2019-04-04

**Authors:** Magdalena Posadzy, Filip Vanhoenacker, Vasiliki Siozopoulou

**Affiliations:** 1 W. Dega Orthopaedic and Rehabilitation University Hospital, Karol Marcinkowski University of Medical Sciences, Poznan, PL; 2 AZ Sint-Maarten and University (Hospital) Antwerp/Ghent, BE; 3 University Hospital Antwerp, Wilrijkstraat, Edegem, BE

**Keywords:** juxtacortical chondroma, phalanx, Cone Beam CT, MRI, Radiography

## Abstract

Juxta-cortical chondroma is a rare cartilaginous tumor originating from the periosteum. On conventional radiography, the lesion typically causes saucerization of the adjacent cortex with well-delineated sclerotic margins. Projection radiography may be less accurate than cross-sectional imaging to demonstrate the precise extent of pressure erosion and bone and soft tissue extent. Although magnetic resonance imaging (MRI) is the imaging technique of choice for further preoperative evaluation, cone-beam computed tomography (CT) may be of additional value. Due to its high spatial resolution, cone-beam CT may detect very tiny matrix calcifications and allows a more precise evaluation of the saucerized cortex at a low radiation dose.

## Introduction

Juxta-cortical chondroma (JCC) is a rare benign cartilaginous tumor originating from the periosteum [1]. Imaging features have been reported previously and are characterized by cortical scalloping with overhanging edges, chondroid matrix, and soft tissue mass [2]. Detection and characterization is usually done by conventional radiographs (CR), followed by magnetic resonance imaging (MRI). This paper aims to report the potential use of cone-beam computed tomography (CBCT) in preoperative evaluation of JCC.

## Case Report

### Case 1

**Figure 1:**
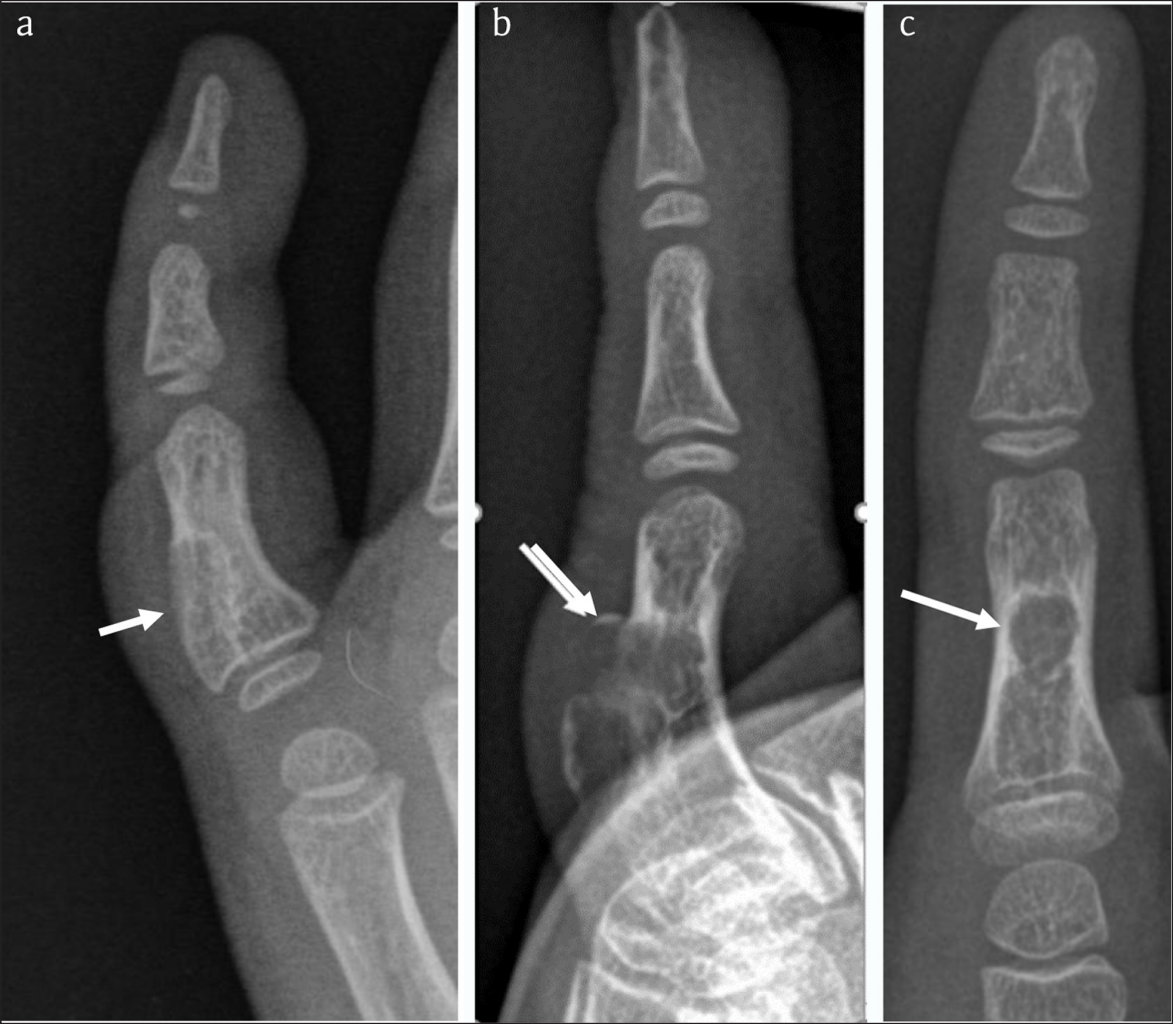
Case 1 – Conventional radiographs of the right fifth finger in a nine-year-old boy. Oblique view taken five years prior to the current admission **(a)**. The lesion (arrow in a) was originally interpreted as a non-ossifying fibroma. Lateral **(b)** and anteroposterior (AP) view at current admission **(c)**. The lesion has grown and consists of two components. The largest juxta-cortical and exophytic part causes pressure erosion of the dorsal cortex, which is thinned and focally destructed with overhanging edges (arrow in b). There is an associated soft tissue swelling. The smaller intramedullary part consists of a well-delineated radiolucent lesion with sclerotic borders. The latter is best seen on the AP view (arrow in c).

**Figure 2:**
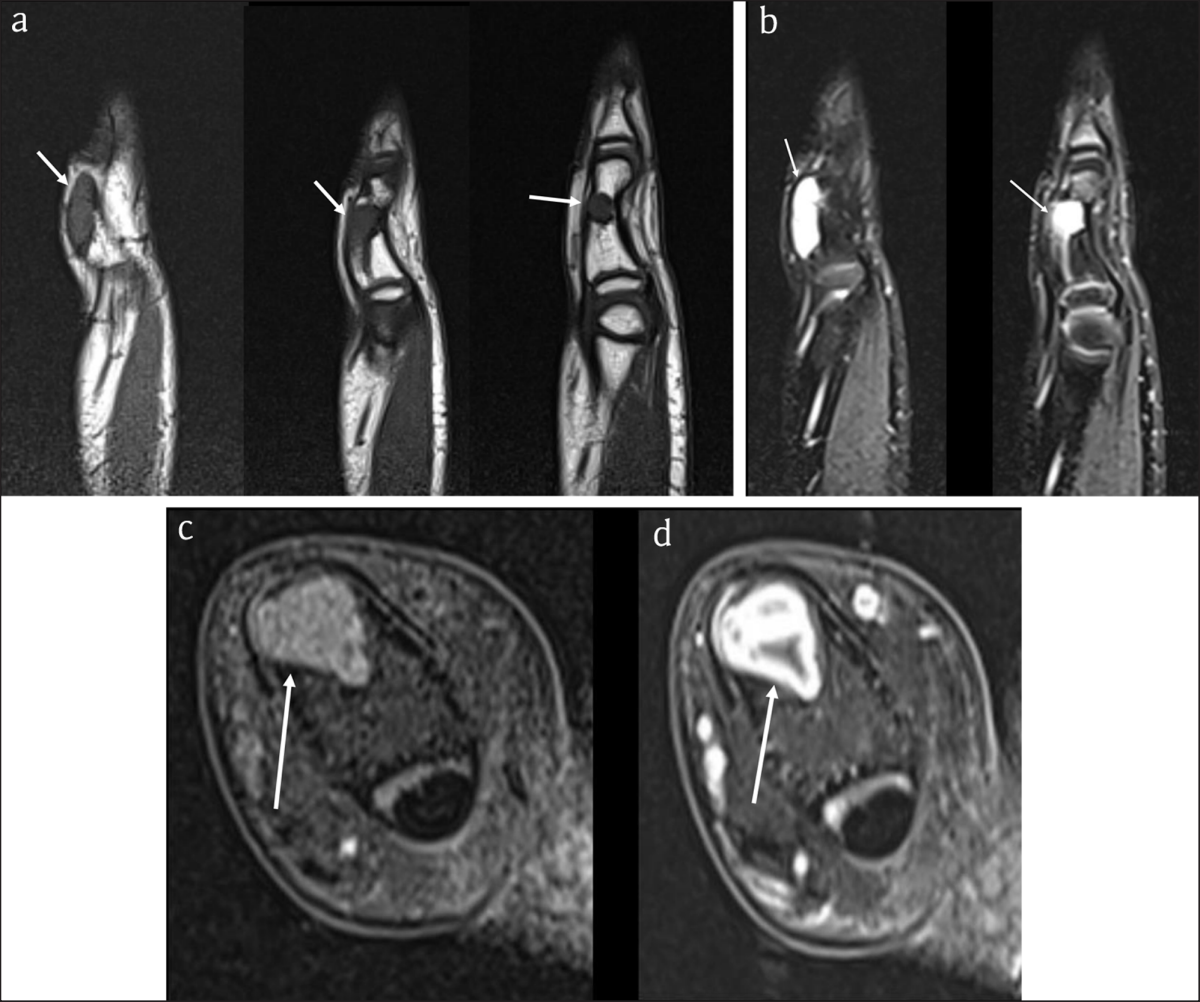
Case 1 – MRI. Sagittal T1-WI (3 adjacent slices) **(a)**. Sagittal fat-suppressed (FS) T2-WI (2 adjacent slices) **(b)**. Axial FS T1-WI **(c)**. Axial FS T1-WI after intravenous administration of gadolinium contrast **(d)**. The lesion is isointense to muscle on T1-WI (arrows in a) and hyperintense on FS T2-WI (arrows in b) and enhances peripherally, in keeping with chondroid matrix (arrow in d).

**Figure 3:**
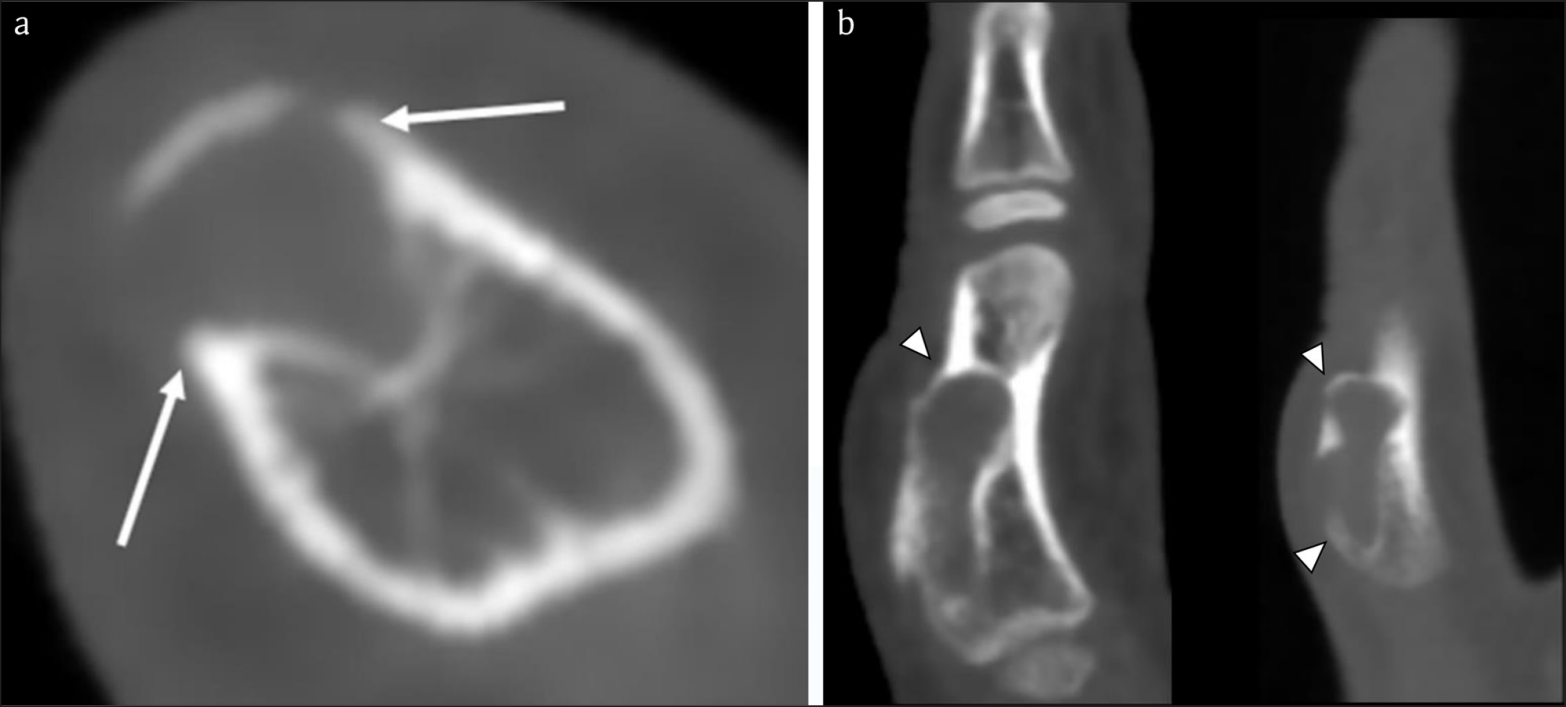
Case 1 – CBCT. Axial image **(a)**. Sagittal reformatted images (2 adjacent slices) **(b)**. Note an expansile radiolucent lesion at the dorsal phalangeal cortex with sclerotic borders (arrows in a). The smaller intramedullary and a larger exophytic components of the lesion are clearly depicted. The cortex is thinned and partially destructed with overhanging edges (arrowheads in b).

A nine-year-old boy presented with a slowly progressive swelling at the dorso-ulnar aspect of the proximal phalanx of the fifth finger of the left hand. CR performed five years previously revealed a well-delineated cortical lesion, originally interpreted as a non-ossifying fibroma (Figure 1a). CR at admission showed lesion enlargement, consisting of two components. The largest juxta-cortically part eroded the dorsal cortex with overhanging bony edges. The smaller rounded intramedullary part was well-delineated with sclerotic borders (Figure 1b–c). Subsequent MRI was performed to evaluate soft tissue and bone marrow involvement. On (FS) T1-weighted images (WI) the lesion appeared isointense to muscle with well-defined borders (Figure 2a) and was hyperintense on T2-WI (Figure 2b). Peripheral enhancement was seen (Figure 2c–d). CBCT revealed cortical saucerization, cortical breakthrough and focal extra-osseous extent of the lesion (Figure 3a–b).

**Figure 4:**
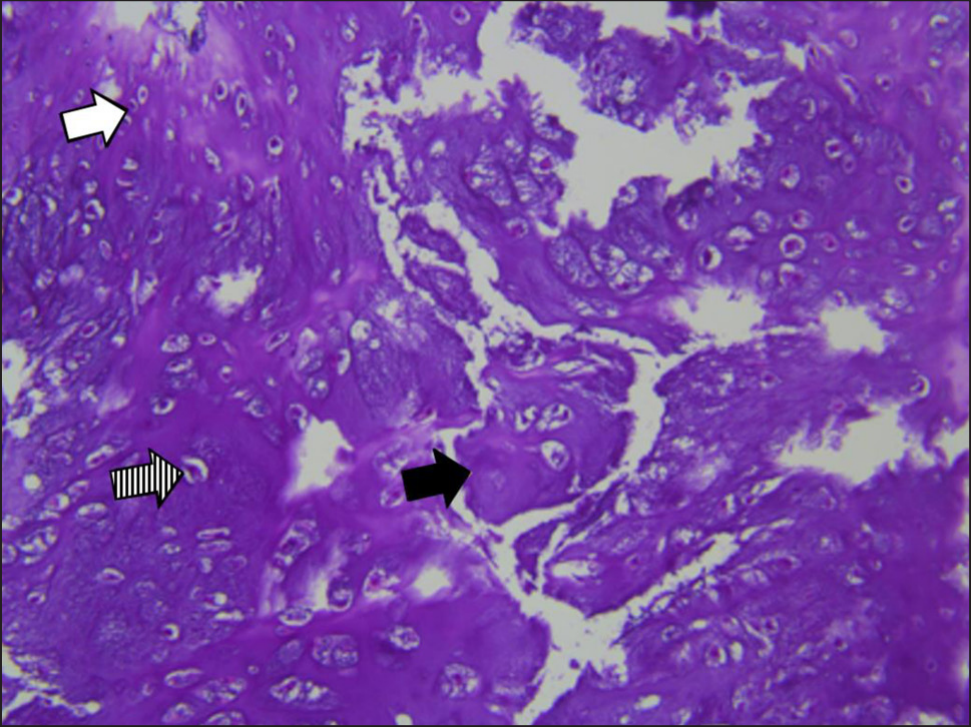
Case 1 – Histopathology of the resected specimen. Hematoxylin eosin staining, 200×:cartilaginous tissue with moderate cellularity. Note chondroid matrix (black arrow) with interspersed small (white arrow) to medium sized (dashed arrow) chondrocytes with no atypia or mitotic activity. There is absence of necrosis.

The lesion was resected followed by bone grafting. Histopathological examination revealed a benign cartilaginous lesion (Figure 4).

### Case 2

**Figure 5:**
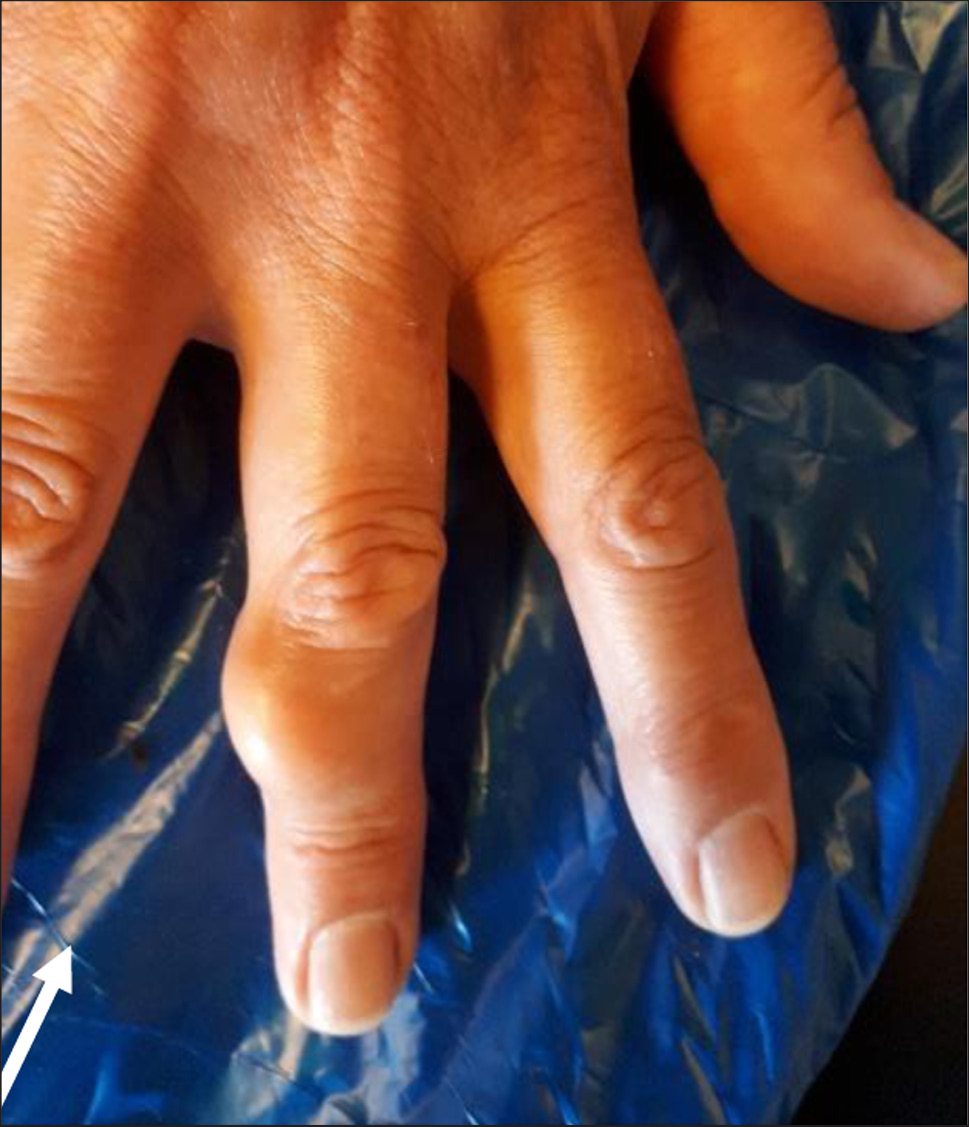
Case 2 – Clinical photograph of the right hand in a 59-year-old woman, showing a swelling at the dorso-ulnar aspect of the third finger (arrow).

**Figure 6:**
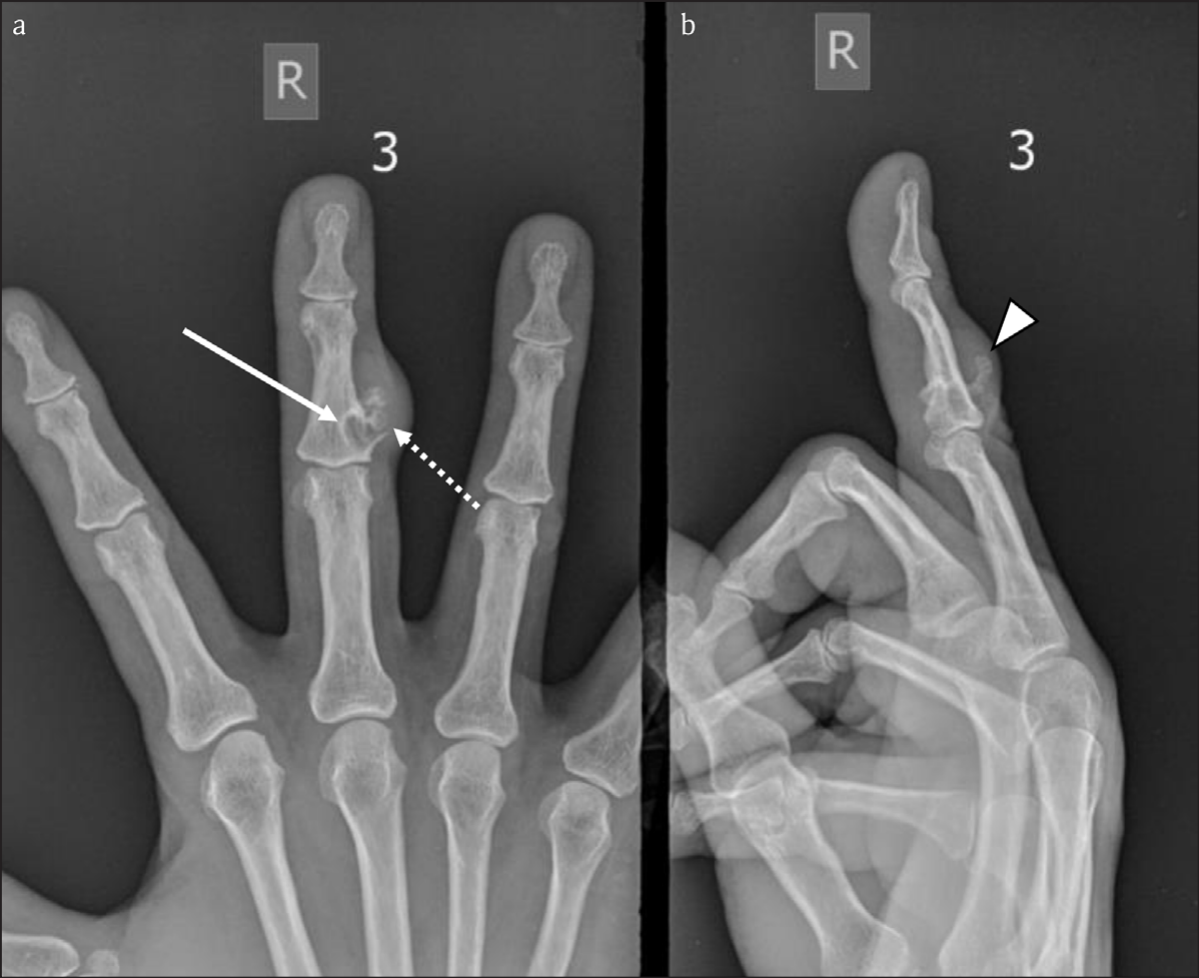
Case 2 – Conventional radiographs of the right third finger. AP **(a)** and lateral view **(b)**. Note a juxta-cortical radiolucent lesion at the dorso-ulnar aspect of the middle phalanx. The smaller intramedullary part is well-delineated and surrounded by a rim of sclerosis (arrow in a). The larger exophytic component consists of a bony protuberance with adjacent soft tissue swelling (arrowhead in b). There is focal breakthrough of the cortex (dotted arrow in a).

A 59-year-old woman presented with a slowly progressive swelling for 12 months at the middle phalanx of the right third finger (Figure 5). CR revealed a juxta-cortical radiolucent lesion, consisting of two components. The smaller intramedullary part was well-delineated and surrounded by a sclerotic rim. The larger exophytic component consisted of a bony protuberance with adjacent soft tissue swelling. Focal cortical breakthrough was present (Figure 6).

**Figure 7:**
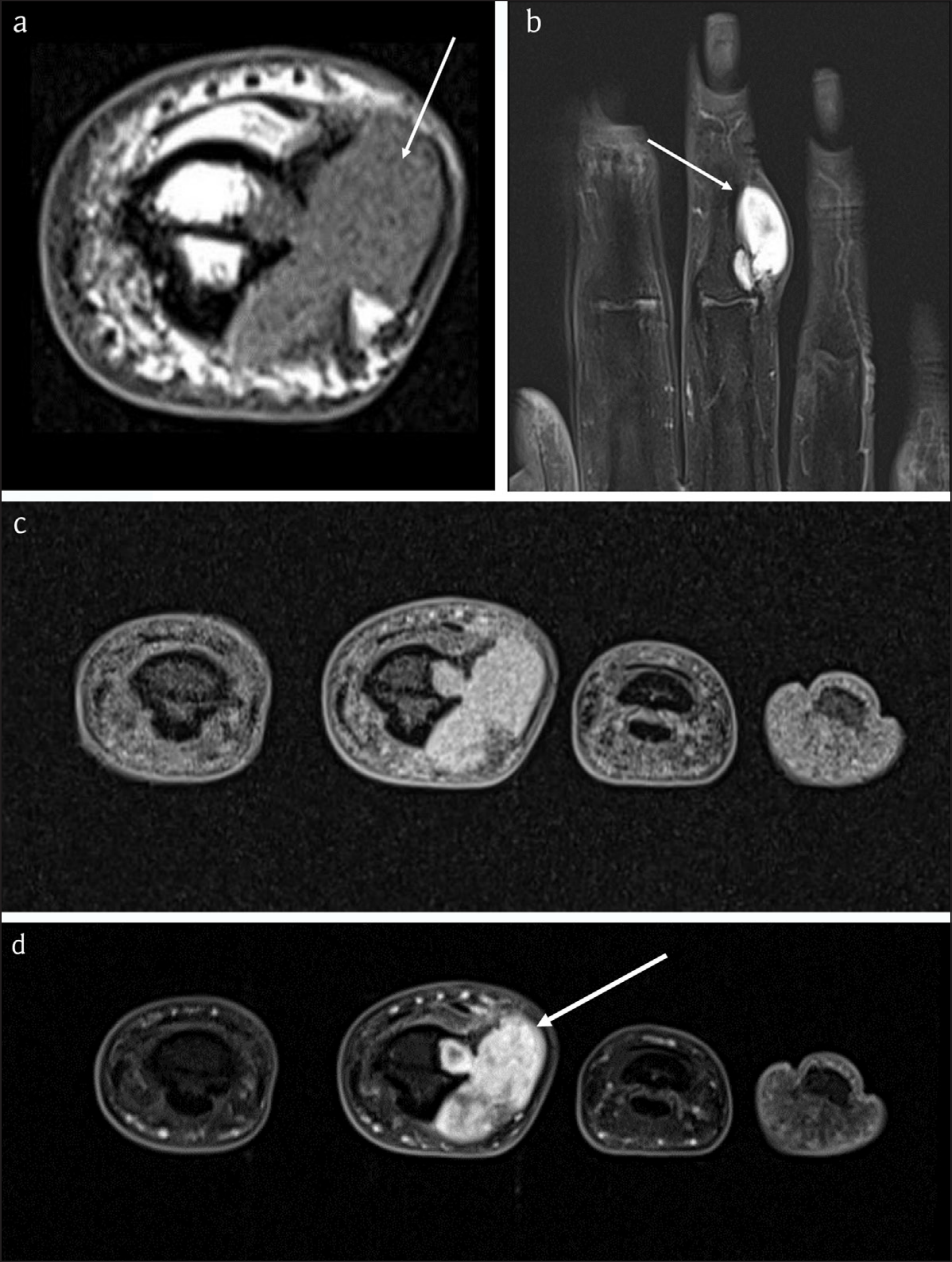
Case 2 – MRI. Axial T1-WI **(a)**. Coronal FS T2-WI **(b)**. Axial FS T1-WI **(c)**. Axial FS T1-WI after administration of gadolinium contrast **(d)**. The lesion is isointense to muscle (arrow in a) and hyperintense on FS T2-WI (arrow in b) with ring-and-arc enhancement (arrow in d), in keeping with chondroid matrix.

**Figure 8:**
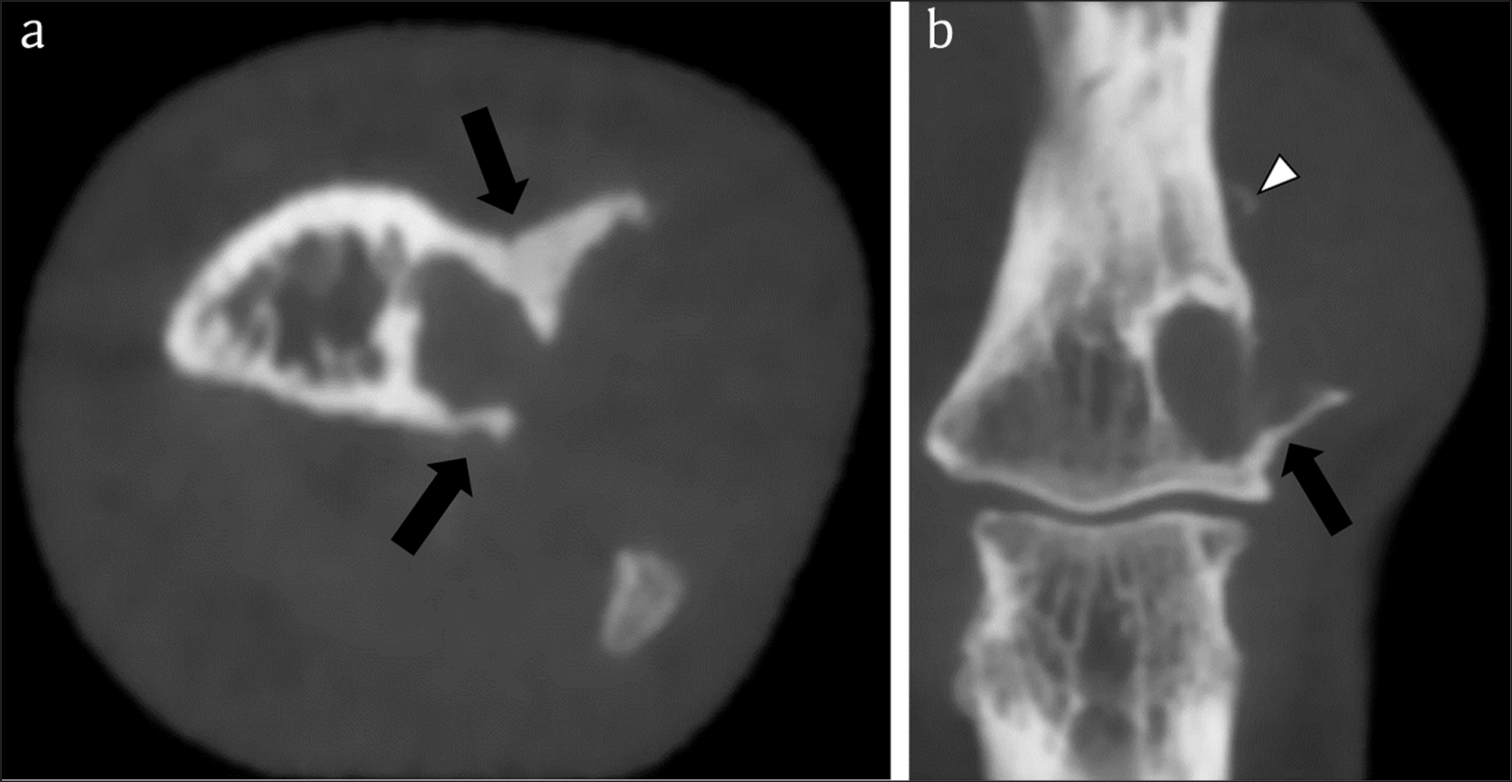
Case 2 – CBCT. Axial image **(a)**. Coronal reformatted image **(b)**. The lesion consists of a smaller intramedullary and a larger juxta-cortical component. The intramedullary part is well-delineated by a rim of peripheral sclerosis, whereas the juxta-cortical part causes scalloping of the outer cortex within overhanging edges (black arrows in a and b). The cortical breakthrough and overhanging margins are far better demonstrated than on CR (see Figure 6). Note a small fleck of calcification (white arrowhead), supporting the hypothesis that the lesion contains chondroid matrix.

MRI confirmed a juxta-cortical lesion with associated intramedullary extension. The lesion was isointense to muscle on T1-WI (Figure 7a) and hyperintense on FS T2-WI (Figure 7b) and showed ring-and-arc enhancement (Figure 7c–d). CBCT revealed an osteolytic lesion with a small intramedullary and a larger juxta-cortical component with very subtle matrix calcifications and cortical saucerization (Figure 8).

**Figure 9:**
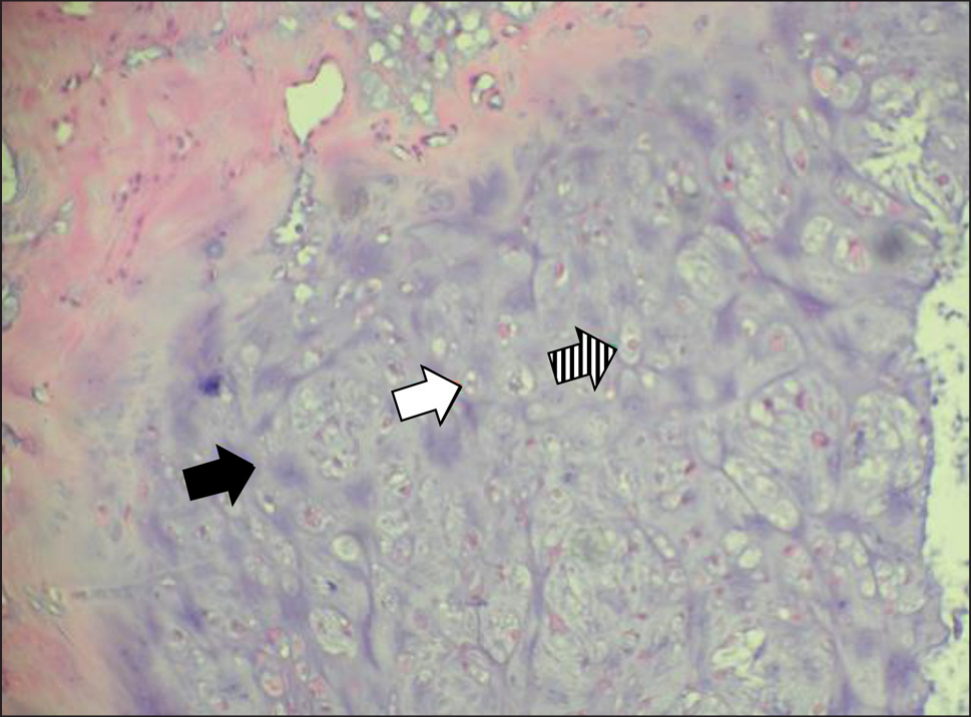
Case 2 – Histopathology of the resected specimen. Hematoxylin eosin staining, 200×: cartilaginous tissue with moderate cellularity. Note chondroid matrix (black arrow) with interspersed small (white arrow) to medium sized (dashed arrow) chondrocytes with no atypia or mitotic activity. There is absence of necrosis.

The lesion was resected and filled-up with bone grafts. Histopathology demonstrated a benign cartilaginous tumor (Figure 9).

## Discussion

Juxta-cortical chondroma, previously designated as periosteal chondroma, is a rare benign chondroid tumor, accounting for 1.3% of cartilaginous tumors [1]. Since the precise anatomical point of origin cannot always be determined, the term “juxta-cortical” is preferred above “periosteal” [3]. As the lesion grows, it may extend on either the outer and inner cortex resulting in a exophytic part with associated soft tissue component and an endophytic part extending within the medullary cavity [1]. Hands and feet are most frequently involved. Other long bones, cervical vertebra, and clavicle may be affected [1,2,4]. Clinically, patients present with a slowly growing mass. It predominantly occurs in the second decade of life, but any age may be involved [5,6].

On CR, the endophytic part of the lesion has a well-defined, sharp sclerotic margin. The exophytic part of the lesion typically causes pressure erosion of the cortex also known as saucerization [7]. These features are indicative of the slow growth of the lesion.

On MRI, the lesion is hypo- or isointense to muscle on T1-WI, is hyperintense on T2-WI, and demonstrates a peripheral or ring-and-arc enhancement pattern, in keeping with chondroid tissue. The juxta-cortical soft tissue component causes pressure erosion with cortex remodelling. Due to its higher spatial resolution than MRI, CT is superior for demonstrating characteristic overhanging bone spicules. Although saucerization is usually visible on CR, superimposition of bone may hamper evaluation of the precise extent. Whereas the shape of overhanging borders may resemble a “Codman’s triangle” on CR, seen in aggressive intramedullary lesions, CT more clearly depicts the well-delineated overhanging shelf of bone around the proximal and/or distal ends of the exophytic component of a benign juxta-cortical lesion [1]. Furthermore, CT is more sensitive than CR and MRI in depiction of subtle intralesional chondroid calcifications.

The main drawback of CT is its high radiation dose. Generally, CBCT has a lower radiation dose in comparison to most multi-detector computed tomography (MDCT) equipment. However, with the use of specialized dose modification, radiation dosage of state-of-the art MDCT may be lowered. Another advantage of CBCT is its compact design and lower cost for installation and maintenance. CBCT was particularly useful in case 2, in which cortical breakthrough and extent of the overhanging bony edges were underestimated on CR.

Histopathologically, JCC is characterized by a cellular hyaline to myxoid cartilage lacking nuclear hyperplasia. Nuclei are small and round with condensed chromatin. Slightly larger nuclei with open chromatin and small nucleoli are common. The cells can be evenly distributed or arranged in small clusters. More than one cell per lacuna as well as binucleated cells can be present but those features are rather occasional. Mitotic activity is absent [1]. Focal calcifications and mucoid matrix degeneration can be observed [2].

The differential diagnosis of JCC include other juxta-cortical lesions such as non-ossifying fibroma (NOF), desmoid, cortical neurofibroma, Nora’s lesion, periosteal chondrosarcoma and periosteal osteosarcoma [2,8]. NOF rarely affects the bones of the hand and a soft tissue component is absent. Nora’s lesion presents as ossified exophytic lesion with intact cortex [1]. Chondrosarcoma rarely affects the phalanges and is generally accompanied by a larger soft tissue component [5]. Histologically, JCC may be misinterpreted as chondrosarcoma if phalangeal location is not taken into account and in case of inappropriate analysis of the radiological semiology [1,8]. In periosteal osteosarcoma, the matrix is osteoid, the lesion tends to be larger with more aggressive growth and periosteal reaction and absence of sclerotic delineation [1,4].

Treatment includes complete tumor excision with subsequent bone graft filling [7,9].

## Conclusion

CBCT may be of additional value to CR and MRI in preoperative characterization and staging of a JCC at a low radiation. Due to the lack of superimposing structures, CBCT may allow more precise interpretation of characteristic radiological features than CR.
